# Rest the brain to learn new gait patterns after stroke

**DOI:** 10.1186/s12984-024-01494-8

**Published:** 2024-10-29

**Authors:** Chandramouli Krishnan, Thomas E. Augenstein, Edward S. Claflin, Courtney R. Hemsley, Edward P. Washabaugh, Rajiv Ranganathan

**Affiliations:** 1 Department of Physical Medicine and Rehabilitation, Neuromuscular and Rehabilitation Robotics Laboratory (NeuRRo Lab), Michigan Medicine, University of Michigan, 325 E Eisenhower Parkway (Room 3013), Ann Arbor, MI 48108 USA; 2 Department of Robotics, University of Michigan, Ann Arbor, MI USA; 3 Department of Mechanical Engineering, University of Michigan, Ann Arbor, MI USA; 4 School of Kinesiology, University of Michigan, Ann Arbor, MI USA; 5 Biomedical Engineering, University of Michigan, Ann Arbor, MI USA; 6 Department of Physical Therapy, University of Michigan-Flint, Flint, MI USA; 7 Department of Biomedical Engineering, Wayne State University, Detroit, MI USA; 8 Department of Kinesiology, Michigan State University, East Lansing, MI USA; 9 Department of Mechanical Engineering, Michigan State University, East Lansing, USA

**Keywords:** Skill acquisition, Error-based learning, Motor task, Hemiparesis, Consolidation

## Abstract

**Background:**

The ability to relearn a lost skill is critical to motor recovery after a stroke. Previous studies indicate that stroke typically affects the processes underlying motor control and execution but not the learning of those skills. However, these studies could be confounded by the presence of significant motor impairments. Furthermore, prior research involving the upper extremity indicates that stroke survivors have an advantage in offline motor learning when compared with controls. However, this has not been examined using motor acuity tasks (i.e., tasks focusing on the quality of executed actions) that have direct functional relevance to rehabilitation.

**Objective:**

Investigate how stroke affects leg motor skill learning during walking in stroke survivors.

**Methods:**

Twenty-five participants (10 stroke; 15 controls) were recruited for this prospective, case-control study. Participants learned a novel foot-trajectory tracking task on two consecutive days while walking on a treadmill. The task necessitated greater hip and knee flexion during the swing phase of the gait. Online learning was measured by comparing tracking error at the beginning and end of each practice session, offline (rest-driven) learning was measured by comparing the end of the first practice session to the beginning of the second, and retention was measured by comparing the beginning of the first practice session to the beginning of the second. Online learning, offline learning, and retention were compared between the stroke survivors and uninjured controls.

**Results:**

Stroke survivors improved their tracking performance on the first day (*p* = 0.033); however, the amount of learning in stroke survivors was lower in comparison with the control group on both days (*p* ≤ 0.05). Interestingly, stroke survivors showed higher offline learning gains when compared with uninjured controls (*p* = 0.011).

**Conclusions:**

Even stroke survivors with no perceivable motor impairments have difficulty acquiring new motor skills related to walking, which may be related to the underlying neural damage caused at the time of stroke. Furthermore, stroke survivors may require longer training with adequate rest to acquire new motor skills.

## Background

Stroke is a major cause of adult disability worldwide, affecting millions of people each year [1, 2]. Common motor impairments after stroke include weakness on one side of the body [3], difficulty coordinating movements [4, 5], and loss of balance [6]. These impairments often result in disabilities that restrict the mobility and independence of stroke survivors in their daily activities, which in turn highlights the need for effective rehabilitation techniques that can improve walking ability. Current approaches to gait recovery after stroke often involve task-specific training with assistive devices and interactive technologies [7, 8]. However, despite their effectiveness, these methods are no more beneficial than conventional rehabilitation in most clinical trials [9–11]. Therefore, there is a critical need for new therapies that can facilitate gait recovery after stroke.

A key to developing effective rehabilitation interventions after stroke is through the application of motor learning principles. Although the importance of incorporating motor learning principles into stroke rehabilitation programs has been repeatedly emphasized [12, 13], there still remains a large gap in our understanding of how learning and rehabilitation processes are interlinked in clinical populations [14]. There is some evidence that acquiring new skills can activate neuroplastic mechanisms in the brain and that the process of learning a new motor skill shares similarities with relearning lost motor skills following a stroke [12, 15]. Therefore, studying motor learning deficits after a stroke can provide a better understanding of the specific mechanisms of neurophysiological recovery, which could aid in the development of more effective interventions.

However, the effect of stroke on motor skill learning is difficult to estimate, as there is limited research on this topic and previous research has yielded conflicting results. For example, some studies suggest that stroke primarily affects the processes underlying motor control and execution, while leaving the learning of motor skills intact [16–19]. However, a recent study revealed that the extent of motor learning deficits following a stroke is dependent on the severity of motor impairment [20]. It is important to note that a major challenge in establishing evidence of learning deficits is that performance deficits can be misinterpreted as learning deficits [21, 22]. This is supported by the observation that error-based learning capacity—learning driven by error relative to a desired action or goal—in stroke survivors is comparable to neurologically intact adults when motor execution deficits are controlled for during the experiment [16, 19]. However, many of these prior studies, for good reasons, have focused on goal or action selection (i.e., where to move to or what movement can achieve the chosen goal) with less emphasis on motor acuity (i.e., the quality of the executed movements) [23]. More importantly, the experimental tasks are often restricted to a single degree of freedom (DOF) movement, thereby making it challenging to generalize these findings to complex multi-DOF movements (e.g., gait) and limiting their functional relevance to rehabilitation.

Another challenge in determining the effect of stroke on motor skill learning is that learning is mediated by both online and offline processes that may be differentially affected by stroke. Specifically, changes in performance can be due to learning during practice or from periods of rest between practice when information is consolidated and committed to long-term memory [24]. Changes due to practice are typically measured by evaluating the change in performance from the beginning to the end of training (i.e., online learning), while consolidation is measured by evaluating changes in performance from the end of training to the beginning of a follow-up session conducted in the following days (i.e., offline gains/learning) [25]. The summation of these two processes can be evaluated by retention of learning, which is measured as a change from the beginning of training to the follow-up session. Previous studies have shown that stroke survivors improve their performance on motor skills following a period of sleepful rest, while uninjured controls reduce their performance. Interestingly, these same learning gains are not observed in stroke survivors who rest for an equivalent time interval without sleep [26–28]. As such, it seems possible that stroke survivors undergoing interventions involving learning may benefit from periods of sleep between practice sessions. Furthermore, it is possible that this advantage in offline gains is masking online deficits in studies that only measure retention. However, as mentioned above, these contributions of offline gains to skill learning in stroke survivors have not been investigated in complex, lower-extremity tasks that have relevance to gait rehabilitation. As a result, it is currently unclear how stroke affects motor learning and whether learning deficits are present in individuals with minimal impairment when performing functional lower-extremity tasks such as walking.

Therefore, the purpose of this study was to evaluate the extent of motor learning deficits in chronic stroke survivors using a functional leg motor skill learning task. To minimize the impact of paresis/weakness on our findings, we specifically recruited stroke survivors with minimal impairment. To comprehensively understand the effect of stroke on motor learning, we examined both online (i.e., changes that occur during practice within the same day) and offline (i.e., changes that occur after practice during periods of no practice between days) learning. To address the issue of task relevance to day-to-day activities, the task required participants to learn a gait pattern that required 30% greater hip and knee flexion during the swing phase, which has been previously shown to be highly relevant in rehabilitation training for addressing stiff knee gait after stroke [29]. We hypothesized that stroke survivors with mild motor impairments would exhibit significant deficits in both online and offline learning and retention of motor skills during walking when compared with uninjured controls.

## Methods

### Participants

A total of 25 adults (10 individuals with stroke and 15 elderly uninjured controls, Table 1) participated in this study. Older adults of similar age to the stroke survivors were recruited as controls to minimize age as a confounding factor in our analysis. This sample size provided us with a power β > 80% to detect statistical significance with a conservative effect size of *n*^*2*^ = 0.3 [30, 31] at a significance level of α = 0.05 (computed in G*power 3.1.9.6, Test family: F tests, ANOVA: Repeated measures, within-between interaction). Participants in the control group were also part of a different study that investigated the effects of aging on motor learning [31]. All participants were right leg dominant based on their preferred leg to kick a ball [32]. Stroke survivors were included in the study if they (1) had a radiologically (CT or MRI) confirmed ischemic or hemorrhagic stroke at least 6 months prior to the study, (2) had no significant cognitive deficits (Mini-Mental State Examination [MMSE] Score ≥ 22), (3) had no documented major sensory or proprioceptive deficits (determined via self-assessment and medical chart review during screening), (4) had no major motor deficits (determined by lower-extremity Fugl-Meyer ≥ 27 or 78% of the total scale of 34 [33]), (5) were able to walk independently with or without assistive devices, (6) had no history of uncontrolled diabetes or hypertension, and (7) had no major orthopaedic issues or range of motion deficits. Control participants were included in the study if they (1) had no significant cognitive deficits (MMSE ≥ 22), (2) had no significant orthopaedic or neurological issues, and (3) had no history major medical conditions, including uncontrolled diabetes or hypertension. We measured the stroke survivors’ lower extremity motor impairment with the lower-extremity Fugl-Meyer scale (LE-FM, 32.1 ± 2.2, range: 27–34, one participant was not measured). All participants were tested at a single laboratory within the University of Michigan and signed a written informed consent prior to participation that was approved by the University of Michigan Human Subjects Institutional Review Board.

**Table 1:** Demographics of participants. Mean ± standard deviation for age, mass, height, MMSE score, lower-extremity Fugl-Meyer (LE-FM), self-selected gait speed, and sleep quality have been reported. The MMSE score can range from 0 to 30. LE-FM score can range from 0 to 34. Sleep quality rated on scale from 0 (best possible) to 10 (worst possible)

Group	Sex	Age (year)	Mass (kg)	Height (m)	MMSE score	LE-FM score	Gait speed	Sleep quality
Stroke	5 females, 5 males	58.3 ± 8.9	76.5 ± 20.5	1.7 ± 0.1	28.3 ± 2.2	32.1 ± 2.2	1.3 ± 0.2	2.5 ± 2.2
Control	10 females, 5 males	65.3 ± 2.9	72.0 ± 13.2	1.6 ± 0.1	29.3 ± 0.8	N/A	1.24*	3.5 ± 2.9

* indicates that data is taken from normative data set (Kasovic et al. 2021 [49], “Normative Data for Gait Speed and Height Norm Speed in ≥ 60-Year-Old Men and Women”)

### Experimental protocol

**Fig. 1:**
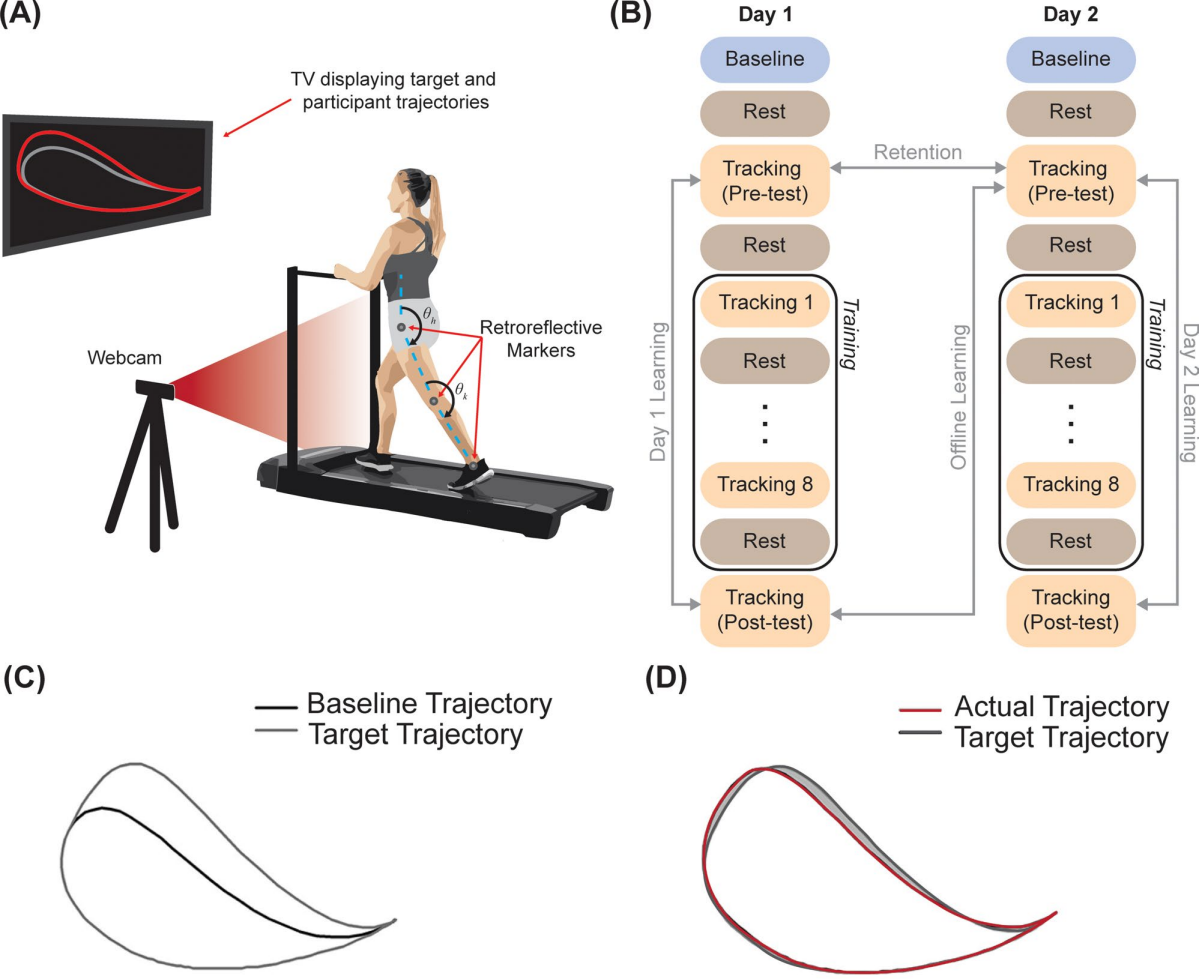
A schematic of the (**A**) experimental set-up and foot-trajectory tracking during treadmill walking, (**B**) experimental protocol, (**C**) participant’s baseline trajectory and their scaled (30%) target trajectory, and (**D**) computation of tracking error represented by the non-overlapping area (shaded in grey)

Participants learned a foot-trajectory tracking task on two consecutive days that were separated by about 24 h (Fig. 1(A)). Participants performed this task with their affected leg while walking on a motorized treadmill that was set to move at a constant speed of 0.89 m/s (2 mph, selected to minimize fatigue and align with previous studies using this paradigm [31, 32, 34, 35]) and wearing the same foot- and leg-wear (i.e., shorts or spandex). The foot-trajectory tracking task required participants to adjust their hip and knee angles during the swing phase of walking to match a target trajectory projected onto a computer monitor placed in front of them. On both days, the experiment consisted of four phases: baseline, pre-test (Pre), training (Tr), and post-test (Post) (Fig. 1(B)). During baseline, participants walked normally on the treadmill for one minute. During the pre-test, the participants performed the foot-trajectory tracking task for one minute and their initial performance on the task was evaluated. Training consisted of repeated practice of the foot-trajectory tracking task. Participants completed eight blocks of practice, with each block lasting one minute and separated by one minute of rest where the treadmill was stopped. In the post-test, the participants again performed the foot-trajectory tracking task for one minute and changes in target-tracking performance were assessed. For stroke participants, the more-affected side was used as the training leg (3 left leg and 7 right leg), and for control participants, the training leg for each participant was determined randomly (7 left leg and 8 right leg). In the post-test, the participants’ final performance was evaluated by assessing their final target-tracking error. Participants were evaluated on the task on two subsequent days so that we could examine the contribution of rest between days to learning.

### Foot-trajectory tracking task

A custom-designed, real-time motion tracking system, developed using LabVIEW 2011 and NI Vision Assistant (National Instruments Corp., Austin, TX, USA), was used for the motor learning task [36]. The system consisted of a camera (C920 Pro HD Logitech Webcam, Logitech, San Jose, CA, USA) and computed the sagittal plane hip and knee kinematics during walking by tracking three 19 mm retroreflective markers positioned on the participants’ greater trochanter, lateral epicondyle of the femur, and lateral malleolus of the ankle. The target template trajectory for the foot-trajectory tracking task was created based on the participants’ sagittal plane hip and knee kinematics data obtained during baseline walking. The target trajectory was generated by scaling (1.3×) the hip and knee angles during swing phase of the normal walking trial and projecting this template in the end-point space, specifically the trajectory of the ankle relative to the hip on the sagittal plane (Fig. 1(C)). This was achieved using the following forward kinematic equation:

$$\:\left[\begin{array}{c}{x}_{a}\\\:{y}_{a}\end{array}\right]=\:\left[\begin{array}{cc}\text{s}\text{i}\text{n}\left({\theta\:}_{h}\right)&\:-\text{s}\text{i}\text{n}({\theta\:}_{k}-{\theta\:}_{h})\\\:-\text{c}\text{o}\text{s}\left({\theta\:}_{h}\right)&\:-\text{c}\text{o}\text{s}({\theta\:}_{k}-{\theta\:}_{h})\end{array}\right]\left[\begin{array}{c}{l}_{1}\\\:{l}_{2}\end{array}\right]$$

Where *x*_*a*_ and *y*_*a*_ are the x and y positions of the ankle lateral malleolus relative to the hip, *l*_*1*_ is the distance between hip and knee markers (i.e., thigh segment), *l*_*2*_ is the distance between knee and ankle markers (i.e., shank segment), *θ*_*h*_ and *θ*_*k*_ are the anatomical hip and knee angles. The target template was smoothed using a Hanning window to prevent abrupt scaling at the beginning and end of the swing phase. The template trajectory was then displayed concurrently with the participant’s actual foot trajectory on a computer monitor positioned in front of the participant. Participants were instructed to try and match the target template trajectory as best as they can during the swing phase of their gait. Additionally, they were asked not to alter the normal gait patterns of the opposite leg that was not involved in the foot-trajectory tracking task.

### Data analyses

The performance on the foot-trajectory tracking task (i.e., how closely the participant’s actual trajectory matched the target trajectory spatially) was evaluated by computing the tracking error for each block. Tracking error was calculated as the difference in area (i.e., non-overlapping area) in pixels between the participant’s actual foot trajectory and the target template trajectory for each stride (Fig. 1(D)). This method of error computation was selected to align with previous work and is useful to prevent positive and negative error cancellation when examining complex shapes [31, 32, 34, 35, 37]. This stride-by-stride tracking error was then expressed as a percentage of the area within the participant’s target template and averaged across strides for each block. Normalizing to each participant’s target template accounts for differences in target templates between participants [31, 32, 34, 35]. For the purposes of this study, four performance metrics were derived from the tracking error data on Day 1 and Day 2: (1) online learning (Day 1), (2) online learning (Day 2), (3) offline learning, and (4) retention (Fig. 1(B)). The amount of online learning on Day 1 (D1) and Day 2 (D2) was evaluated by comparing the tracking error during Pre blocks on Day 1 (D1-Pre) and Day 2 (D2-Pre) to Post blocks on Day 1 (D1-Post) and Day 2 (D2-Post), respectively. The amount of offline learning was evaluated by computing the difference in tracking error during the Pre block on Day 2 (D2-Pre) from the Post block on Day 1 (D1-Post). It is important to note that the offline gain metric is measuring the change in performance between days and is therefore examining the contribution of prolonged rest to learning. The amount of retention was evaluated by comparing the tracking error during the Pre block on Day 1 (D1-Pre) to Pre block on Day 2 (D2-Pre).

### Statistical analysis

All statistical analyses were performed in IBM SPSS for Windows Version 27 (SPSS Inc., Chicago, IL). A two-sample t-test was used to determine if there were any initial performance differences between the two groups. To evaluate if stroke affected the online learning and retention processes, we tested the differences in the amount of online learning (changes in tracking error on Day 1 and Day 2: D1-Post relative to D1-Pre and D2-Post relative to D2-Pre) and retention (initial tracking error on Day 2 relative to initial tracking error on Day 1: D2-Pre relative to D1-Pre) between the stroke and the control participants using repeated measures analysis of covariance (ANCOVA) with block as within-subject factor, group as between-subjects factor, and the appropriate Pre block as the covariate (e.g., D1-Pre was used as a covariate for D1 online learning and retention and D2-Pre was used as a covariate for D2 online learning). To evaluate if stroke affected the consolidation process, we tested the differences in the amount of offline learning (changes in tracking error from the end of Day 1 to the beginning of Day 2: D2-Pre – D1-Post) between the stroke and the control participants using a one-way analysis of variance (ANOVA) with group as the between-subjects factor. These analyses are depicted graphically in Fig. 1(B). A significant interaction effect was followed by appropriate post-hoc analysis with Sidak correction. All analyses used a significance level of $\:\alpha\:$ = 0.05.

### Robustness check analysis

Robustness checks were also performed by evaluating the results in relative terms (i.e., % baseline) and via exponential curve-fitting in addition to the above method [38]. For the relative analysis, we normalized the tracking error as a percentage of their respective baseline values (e.g., Day 1 online learning = [D1-Post/D1-Pre] × 100; offline learning = [D2-Pre/D1-Post] × 100) and compared those values between groups using two-sample t-tests with bootstrapping (10,000 iterations). For the curve-fitting analysis, we used a custom MATLAB program (R2019b, MathWorks, Natick, MA, USA) to fit an exponential function to each participant’s performance using a trust-region-reflective least-squares algorithm and the following expression.

$$\:\underset{\text{a},\text{b},\text{c}}{\text{min}}{\sum\:}_{i=0}^{9}{\left(y\left(i\right)-\stackrel{-}{y}\left(i\right)\right)}^{2}\:\:;\:\:\:\:\:\:\:\:\:\:\:\:\stackrel{-}{y}\left(i\right)=a{\text{e}}^{-bx\left(i\right)}+c$$

Here, $\:{y}$ and $\:\stackrel{-}{y}$ are the participant’s measured and estimated tracking error, respectively, *x*(*i*) is the *i*th experimental block (e.g.,* x*(0) = Pre, *x*(1) = TM-1, etc.), and *a*,* b* and *c* are function coefficients. In the context of this study, coefficients *b* and *c* can be thought of as the learning rate and asymptote, respectively, and *a* + *c* can be thought of as initial error. During fitting, the following bounds were applied on each coefficient to prevent convergence on unreasonable local minima: *a*$\varepsilon$ [min(*y*) – min(*y* [8], *y* [9]), *y* [1] – min(*y* [8], *y* [9])], *b*$\varepsilon$ [0, 5], and *c*$\varepsilon$ [min(*y* [8], *y* [9]), max(*y* [8], *y* [9])]. We then repeated the ANCOVAs used in our primary statistical analyses (e.g., comparing D1-Pre to D1-Post with D1-Pre as a covariate) with the predicted error values and the coefficients. In the latter analysis of the coefficients, *a* + *c* was treated as the initial error and the covariate on each day, and $\:c$ was treated as the final error. To compare the learning rates (*b*) between groups, we also performed a one-way between-subjects ANOVAs on the values of *b* between groups on each day (note that ANOVA was used instead of ANCOVA for analysis of *b* because there is no appropriate covariate for this term). A significance level of α = 0.05 was used for all statistical analyses with appropriate post-hoc comparisons with Sidak adjustments.

## Results

### Day 1 online learning

**Fig. 2:**
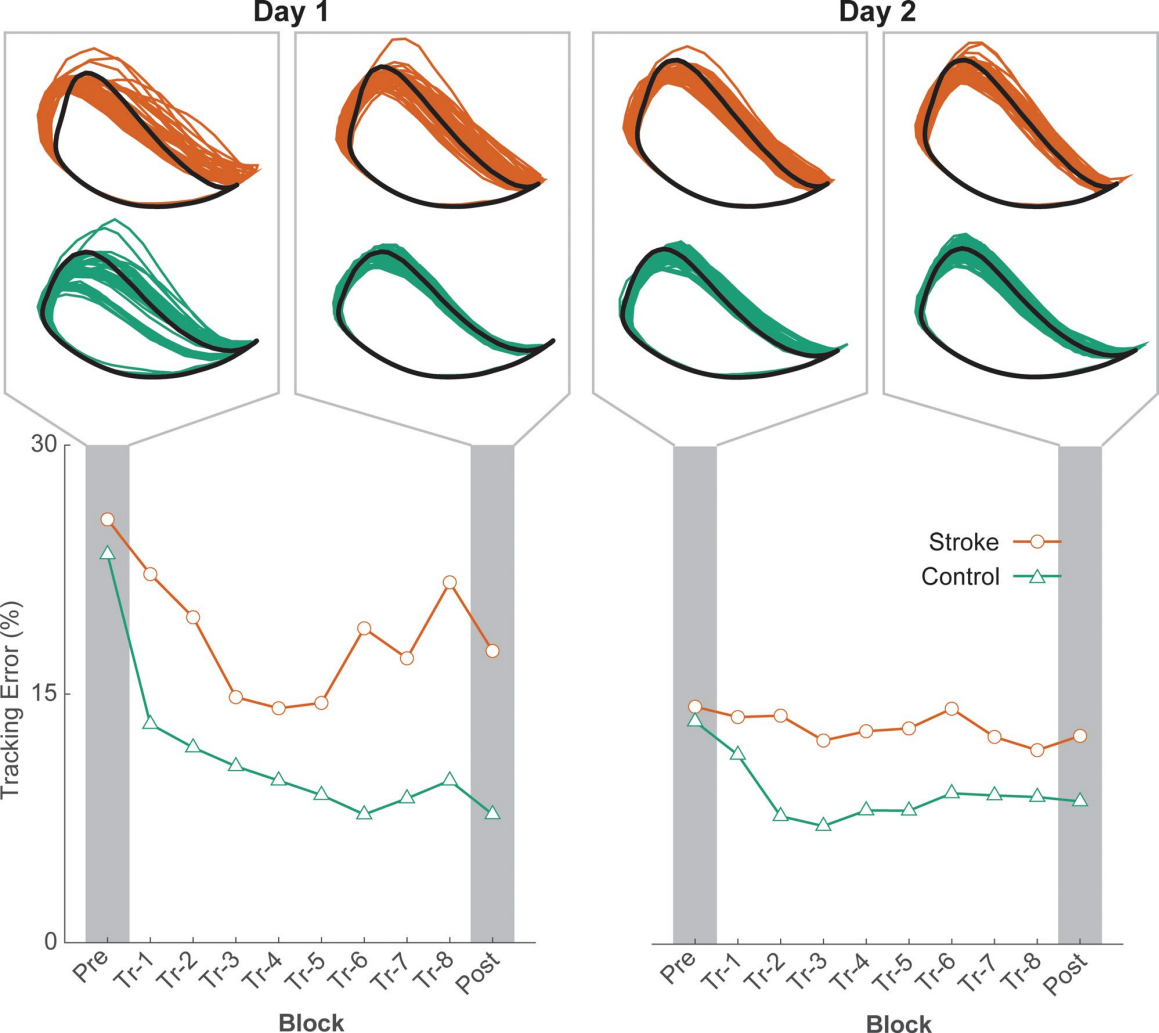
A representative example of participants’ tracking error in each group on Day 1 (left) and Day 2 (right)

**Fig. 3:**
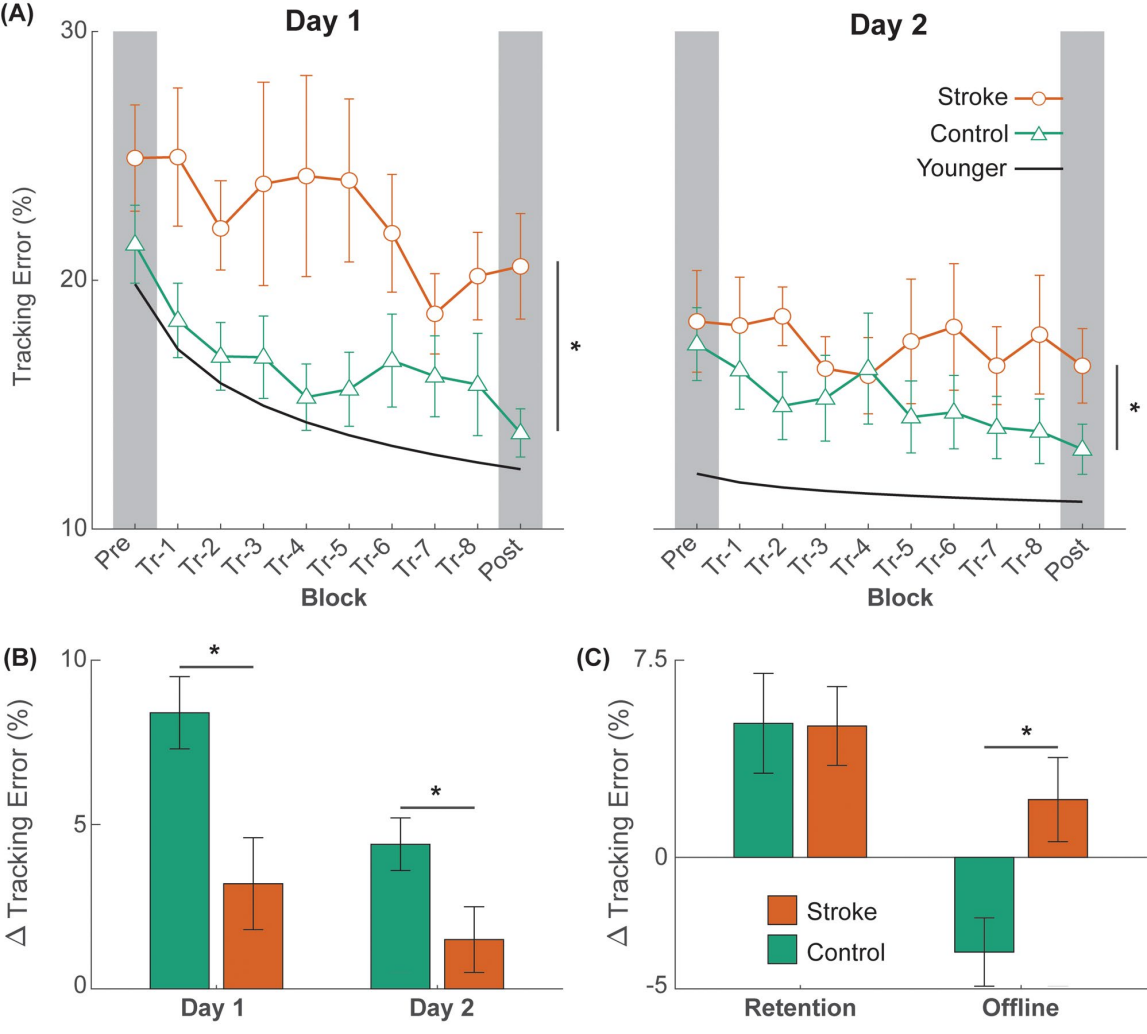
(**A**) The average trajectory tracking error in each group on Day 1 (left) and Day 2 (right). For comparison purposes, we provide data (power-fit curve of the mean data) from young, uninjured adults taken from a previous publication [31]. (**B**) Bar plots showing online differences in learning between the stroke and the control group. (**C**) Bar plots showing differences in the amount of retention and offline gains between the stroke and the control group. Data for online learning and retention are shown as marginal mean changes (Δ) in tracking error. The error bars denote the standard error of the mean and asterisks (*) denotes statistical significance (*p* < 0.05). Positive values indicate improvements in performance

**Fig. 4:**
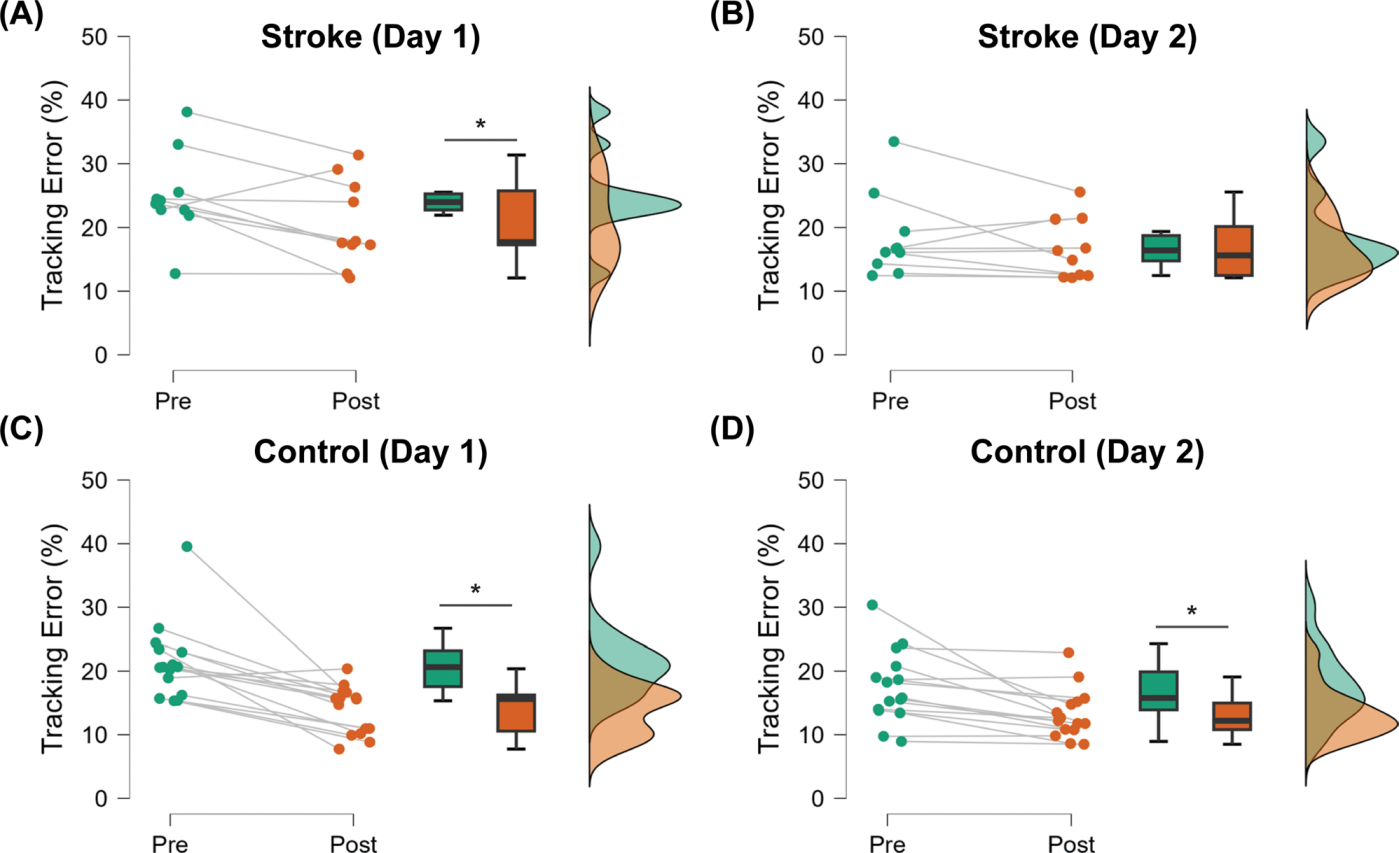
Raincloud plots showing distributions of normalized tracking error before (Pre) and after (Post) training in stroke survivors [top panel, (**A**) and (**B**)] and controls [bottom panel, (**C**) and (**D**)] on both days

Performance of a typical participant from each group is shown in Fig. 2, and average group performance on the tracking task across each block on days one and two is shown in Fig. 3. While there were no initial differences in tracking error between groups (t_1,23_ = 1.338; *p* = 0.194), there was a significant block × group interaction effect on the amount of online learning on Day 1 (F_1,22_ = 7.755; *p* = 0.011). Post-hoc analysis indicated that although both groups improved on tracking performance with practice on Day 1 (stroke: Δ = 3.2 ± 1.4%, *p* = 0.033; control: Δ = 8.4 ± 1.1%, *p* < 0.001; Figs. 3B and 4), the amount of tracking error at the end of practice on Day 1 was greater in stroke survivors when compared with the control group (19.6 ± 1.4% vs. 14.5 ± 1.1%, *p* = 0.033; Figs. 3B and 4). Note that the younger adult curve in Fig. 3 depicts data from a previous study using the same paradigm [31] to provide context in age-related effects on motor learning.

### Day 2 online learning

There was a significant block × group interaction effect on the amount of online learning on Day 2 (F_1,22_ = 4.757; *p* = 0.040; Figs. 3B and 4). Post-hoc analysis indicated that although the control participants improved on tracking performance with practice on Day 2 (Δ = 4.4 ± 0.8%, *p* < 0.001; Figs. 3B and 4), the stroke participants did not (Δ = 1.5 ± 1.0%, *p* = 0.165; Figs. 3B and 4). The amount of tracking error at the end of practice on Day 2 was greater in stroke survivors when compared with the control group (16.3 ± 1.0% vs. 13.4 ± 0.8%, *p* = 0.046; Figs. 3B and 4).

### Retention

**Fig. 5:**
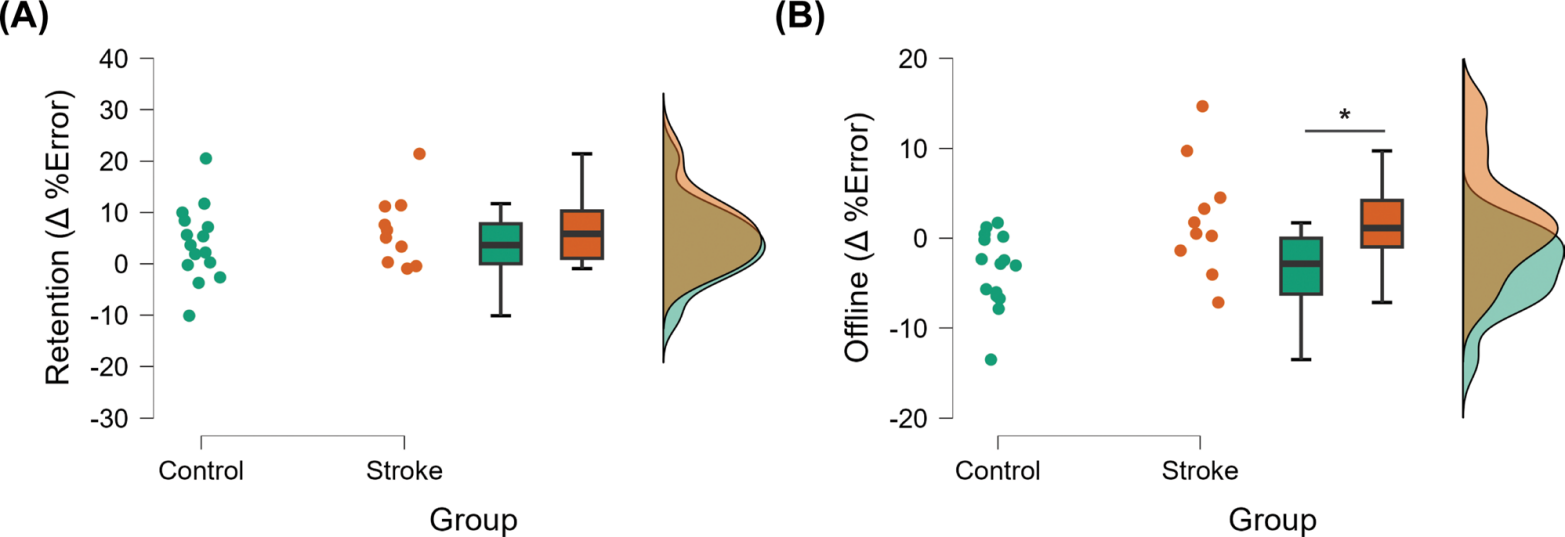
Raincloud plots showing distributions of (**A**) retention (computed as changes in normalized tracking error from Pre block on Day 1 to Pre block on Day 2) and (**B**) offline gains (computed as changes in normalized tracking error from Post block on Day 1 to Pre block on Day 2) in stroke survivors and controls

Retention of performance of the tracking task is shown in Figs. 3C and 5A. There was a significant effect of block on the amount of retention after training (F_1,22_ = 5.403; *p* = 0.030). On average, there was a 5.0 ± 1.5% decrease in tracking error from D1-Pre to D2-Pre in stroke survivors and a 5.1 ± 1.9% decrease in tracking error from D1-Pre to D2-Pre in the control participants. However, there was no group or block × group interaction effect on the amount of retention after training.

### Offline gains

Offline changes in the performance of the tracking task are shown in Figs. 3C and 5B. There was a significant effect of group on the amount of offline gains in motor performance (F_1,23_ = 7.602; *p* = 0.011). On average, stroke survivors experienced a 2.2 ± 1.6% reduction in tracking error from the end of Day 1 to the beginning of Day 2 (indicating offline gains), whereas control participants had a 3.6 ± 1.3% increase in tracking error during the same period (indicating offline loss).

### Robustness check

#### Relative error check

There was a significant difference between groups in the amount of online learning on Day 1 (stroke: 83.6 ± 6.5%, control: 66.7 ± 4.8%, mean difference = 16.9 ± 7.5%, 95% bootstrapped confidence interval = 2.1–32.9%, *p* = 0.045), but the differences in the amount of online learning on Day 2 barely missed statistical significance (stroke: 93.2 ± 6.1%, control: 78.6 ± 4.4%, mean difference = 14.6 ± 7.4%, 95% bootstrapped confidence interval = − 0.1–29.1%, *p* = 0.066). There was also a significant difference between groups in the amount of offline learning (stroke: 92.7 ± 8.0%, control: 127.7 ± 8.1%, mean difference = − 35.1 ± 11.0%, 95% bootstrapped confidence interval = − 56.9–−12.9%, *p* = 0.007) but no difference between groups in the amount of retention (stroke: 75.6 ± 6.7%, control: 84.2 ± 7.4%, mean difference = − 8.6 ± 9.6%, 95% bootstrapped confidence interval = − 28.1–10.2%, *p* = 0.397).

#### Exponential-fit check

**Fig. 6:**
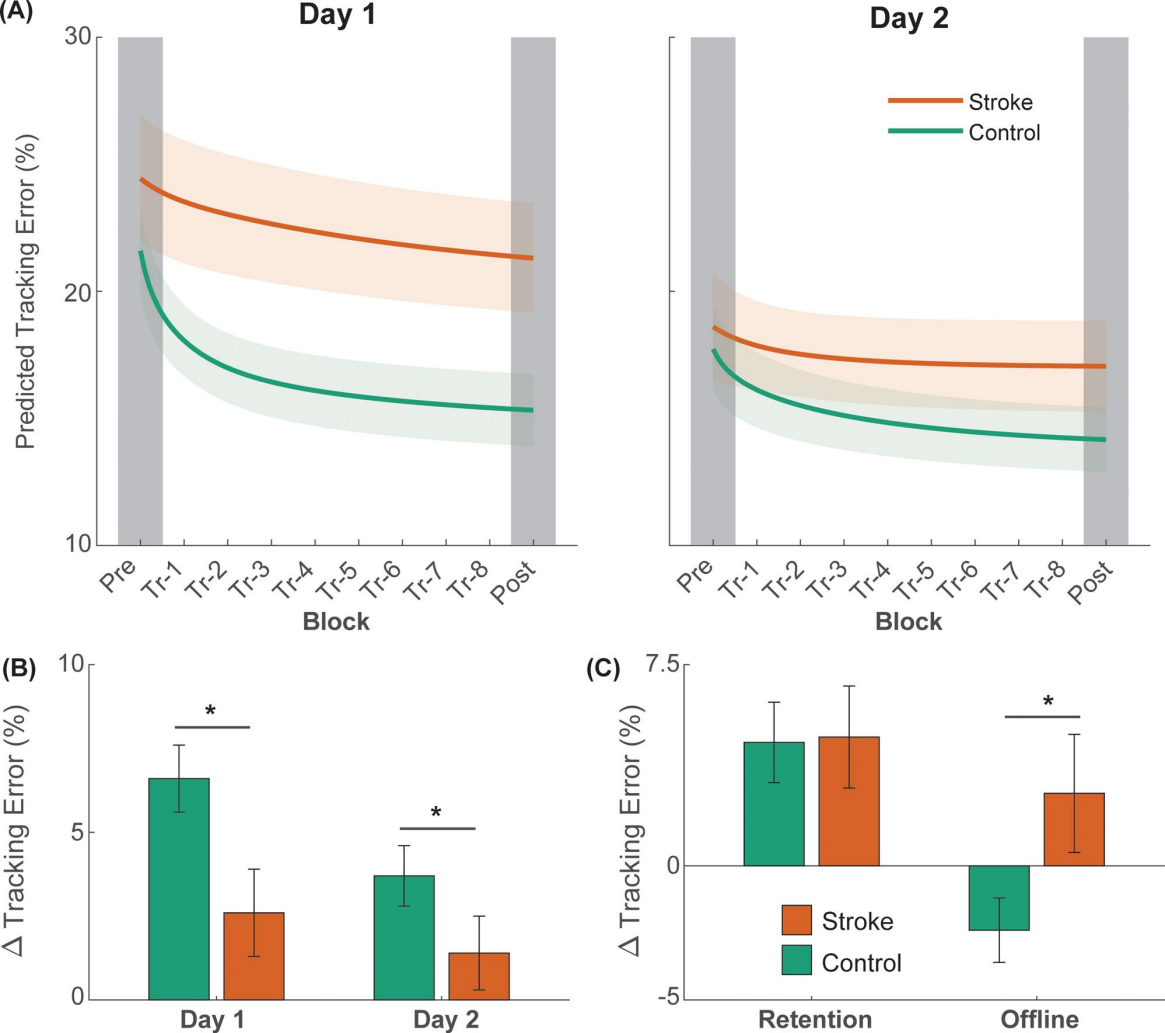
(**A**) Average predicted tracking error in each block on Day 1 (left) and Day 2 (right) using exponential functions fit to participants’ tracking error. Here, the shaded regions represent the standard error of each mean curve. (**B**) Bar plots showing online differences in predicted learning between the stroke and the control group. (**C**) Bar plots showing differences in the amount of predicted retention and offline gains between the stroke and the control group. Data for online learning and retention are shown as marginal mean changes (Δ) in tracking error. The error bars denote the standard error of the mean and asterisks (*) denotes statistical significance (*p* < 0.05). Positive values indicate improvements in performance

The results of the curve-fitting analysis were similar to those of the primary analysis and the first robustness check using relative changes (see Supplementary Material for quantitative results: https://osf.io/hzne3/). Specifically, we found that stroke survivors learned to a lesser extent than controls on Day 1 and Day 2 (Fig. 6(A-B)). There were no between-group differences in retention, but the stroke survivors had greater offline gains than controls (Fig. 6(C)). These robustness checks indicate that our findings were generally robust to different analyses, however the curve-fitting approach increased data variability, resulting in some metrics to fall below significance.

## Discussion

The objective of this study was to examine the extent of motor learning deficits in stroke survivors with low impairment using a functional, lower-extremity task that focused on motor acuity during gait. We focused on motor acuity because the bulk of literature examining motor learning following stroke has focused on the stroke survivor’s ability to select the right action (e.g., amplitude, order in sequence) rather than their quality of movement execution (e.g., kinematics, variability) [23]. Furthermore, learning movement quality is critical for lower extremity tasks, especially in the context of rehabilitation where the quality of a stride is stressed rather than simply its completion [39]. Accordingly, stroke survivors and neurologically intact controls practiced walking on a treadmill with a new gait pattern in two separate sessions. To perform the new gait pattern, participants matched an ankle trajectory that necessitated 30% more hip and knee flexion during the swing phase. We found that stroke survivors showed a lower reduction in tracking error on both days (i.e., online learning deficits) when compared with the control group, who also are known to exhibit learning deficits due to the normal aging process (see Fig. 3 for comparison of both groups to cohort of young, uninjured controls taken from a previous study [31]). Another key finding was that stroke survivors showed offline performance gains between days while the control group showed offline performance loss, indicating that although stroke survivors have lower ability in online learning, they have an advantage in offline learning during a period of rest. Although our study was limited by a small sample size, our participants were quite homogenous in terms of impairment level, and our findings were consistent regardless of how the analyses were performed (i.e., absolute vs. relative learning), indicating the robustness of our results.

The first notable finding from this study is that although stroke survivors were able to learn the task and improve with practice, they showed less improvement in their tracking error as compared with the control group on both days. This finding indicates that stroke survivors have online motor learning deficits when compared with neurologically intact adults, which agrees with some prior studies [40, 41] but differs to some extent from other existing motor learning literature [16–19, 42]. It is likely that this divergence from previous literature results from our examination of mildly impaired individuals using a multi-DOF, functionally relevant motor acuity task during gait. For example, recent research suggests that reinforcement learning was impaired early after the stroke but not in the chronic phase, whereas error-based learning was unaffected after stroke at either time point when compared with controls [16]. However, our study indicates that online motor learning deficits are present even in chronic stroke survivors with minimal motor impairments when compared with controls. A key distinction between the two studies that could explain this discrepancy is the differences in the learning paradigm (skill learning in our study vs. visuomotor adaptation in the previous study). Further, gait is a highly practiced movement involving automatic processes such as balance and posture, and therefore invokes both conscious (e.g., corticospinal) and automatic (e.g., extrapyramidal) motor control pathways [43]. For participants, learning a novel gait pattern requires making conscious alterations to this highly practiced movement, which could influence both these conscious and automatic pathways. On the contrary, non-functional upper-extremity tasks are generally unpracticed motions that do not impact balance and posture and are therefore more likely governed primarily by the conscious motor control pathways [44]. Because these tasks likely require modulation of different motor control pathways, it is possible that they involve different learning mechanisms. Furthermore, even minimally impaired stroke survivors often have diminished balance that can influence their ability to prevent falls [45]. Therefore, it is possible that the observed learning deficits arose from stroke survivors’ resistance to deviate from their current gait pattern and balance on one leg to perform the necessary exploration of the motor control task space, which is necessary to learn a motor skill [46]. 

It is possible that some of the observed learning deficits could be attributed to stroke characteristics. For example, lesions in the prefrontal cortex could have a greater impact during early stages of learning and on motor consolidation processes [47, 48]. Similarly, lesions involving the extrapyramidal systems (e.g., basal ganglia) can disrupt implicit and explicit learning processes [47]. Unfortunately, lesion location information was not evaluated for this study, therefore it is difficult to determine if the observed results could be attributed to damage to specific pathways. Furthermore, other factors that interfere with performance, such as fatigue, differences in preferred gait speed, and affected side, could have contributed to this group difference in learning. However, these factors most likely had a minimal impact because of the following: (1) participants walked at a slow speed and received adequate rest between blocks to prevent fatigue, (2) treadmill speed was sufficiently lower than preferred gait speed for both groups and stroke participants’ gait speed was comparable to controls (Table 1 [49]), and (3) our previous studies with this paradigm have shown that learning is not affected by limb dominance [32]. Future studies should examine stroke-related learning deficits in walking tasks to fully assess the extent of stroke’s effect on learning.

**Fig. 7:**
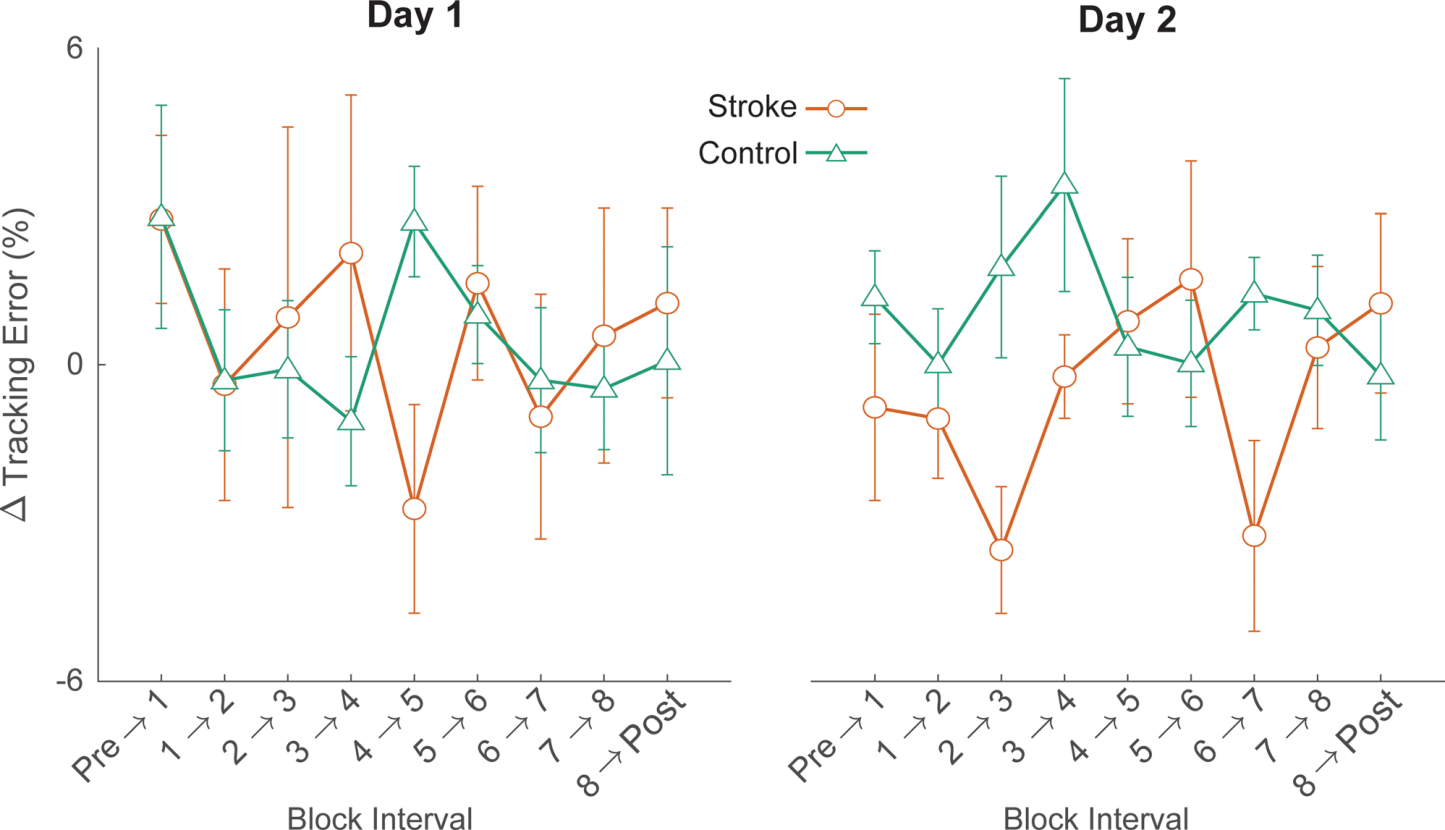
Average micro-offline learning between each block on Day 1 (left) and Day 2 (right). Here, micro-offline learning is defined as the difference in tracking error at the beginning of a block and the error at the end of the previous block. Error bars represent standard error of the mean

Another interesting finding was that despite the deficits in online learning observed in stroke survivors, we did not detect any differences in skill retention between the groups. This occurred because of the differences in offline learning between groups—stroke survivors exhibited offline performance gains whereas the control group exhibited an offline performance loss. This finding aligns with previous research, highlighting the complex interplay between neurological damage and motor learning processes post-stroke [12, 50]. One potential mechanism that could explain decreased ‘online’ learning is ‘reactive inhibition’— the process where performance worsens over repeated executions of the task and improves with breaks [51]. Stroke survivors could exhibit increased reactive inhibition, which interferes in subsequent target-matching and thus decreases their online gains, but when the reactive inhibition dissipates, they “catch up” with the other group. This is typically observed in “massed” vs. “distributed” practice effects – the massed group shows poor online learning but then huge gains over the break [51]. To examine this issue further, we performed an exploratory analysis to determine if stroke survivors also demonstrated offline learning advantages over shorter rest periods. To address this, we computed micro-offline learning for each rest period, defined as the difference between the average tracking error on the first five strides of a target-matching block and the last five strides of the preceding block [52, 53]. Interestingly, there did not appear to be any systematic difference between groups in micro-offline learning (Fig. 7), suggesting that offline learning advantages only appear following longer periods of rest that include sleep.

In our study, it is possible that offline learning could be treated as a measure of sleep-dependent memory consolidation —a process of the central nervous system where recent memory traces are committed to long-term memory during rest—although this should be verified with future experiments with another group that undergoes matched intervals of rest without sleep [54]. Existing research examining this process has shown that stroke survivors demonstrate greater motor performance in upper extremity motor tasks following a period of sleep as compared with an equivalent period of wakefulness, but the same is not true for neurologically intact individuals [27, 28, 48, 55, 56]. Our results directly align with these prior findings and extend them to a functional lower-extremity task. This phenomenon may reflect heightened neural plasticity or alternative neural pathways recruited to compensate for damaged regions wherein post-stroke plasticity facilitates continued skill acquisition (or retention of skills acquired) during rest. Importantly, these findings indicate that stroke-induced neuroplastic changes can also lead to functionally beneficial adaptations apart from the commonly recognized maladaptive processes. Indeed, the observed online learning deficits in conjunction with the offline learning gains could evidence the detriments and benefits of the same repair process; albeit the precise mechanisms underlying these beneficial adaptations are not clear.

Several additional mechanisms could potentially explain why individuals with stroke may exhibit enhanced offline motor learning compared with older controls who do not demonstrate the same phenomenon. First, the brain undergoes significant neuroplastic changes after a stroke as it attempts to reorganize and compensate for the damaged areas [22]. It is possible that these neuroplastic changes enhance the brain’s ability to consolidate and retain motor memories during rest or sleep, leading to improved offline motor learning [50]. Second, stroke survivors often develop compensatory mechanisms (e.g., increased reliance on the premotor cortex or other undamaged regions) to overcome motor deficits [57, 58]. During rest periods, these compensatory mechanisms may be reinforced or optimized, resulting in enhanced offline motor learning [59]. Older controls, who have not experienced neurological damage, may lack the same need for compensatory mechanisms, and therefore, do not exhibit the same enhancement in offline motor learning. Third, stroke can disrupt normal sleep architecture, leading to alterations in sleep stages and patterns [60, 61]. Some studies suggest that certain sleep stages, particularly REM and slow-wave sleep, are crucial for motor memory consolidation [27, 62]. Changes in sleep architecture or enhanced sleep due to pharmacological effects (e.g., gabapentin improves slow wave sleep [63] and total sleep time [64]) post-stroke may create a more conducive environment for offline motor learning compared with older controls. Indeed, the stroke survivors who participated in our study reported superior sleep quality as compared to the controls (Table 1). Finally, aging is associated with decreased neuroplasticity due to alterations in GABAergic activity [65–67], which may impact the brain’s ability to learn and consolidate motor memories during sleep and may be offset by neuroplastic mechanisms following the stroke. It is to be noted though that the differences in online and offline learning observed in this study occurred despite participants being in the chronic period of stroke recovery (i.e., when changes in neuroplasticity are believed to have plateaued), indicating that the enhancement in offline learning could be a sustained phenomenon. Overall, one or many of the above mechanisms likely contributed to the observed differences in offline motor learning between individuals with stroke and older controls. However, it is important to note that retention was only evaluated after one day, so it is unclear if offline learning would have led to improved retention following the second day of training. Therefore, further research is needed to fully elucidate the underlying mechanisms and their implications for stroke rehabilitation.

The results of this study have meaningful implications for post-stroke gait rehabilitation. First, we found that stroke survivors learned to a lesser extent than neurologically intact individuals over the same period. Therefore, it is likely that, when learning or relearning a motor skill following a stroke, stroke survivors will need more practice to achieve similar performance levels as uninjured controls. This would also imply that interventions involving motor learning in stroke survivors should incorporate longer training than in neurologically intact adults. Additionally, we also found that stroke survivors demonstrate learning advantages over uninjured persons over a period of rest involving sleep. Therefore, interventions involving motor learning could likely improve outcomes if they include breaks between sessions where stroke survivors have the opportunity to rest and consolidate learning from practice. However, future research is necessary to fully elucidate the extent of learning deficits in stroke and how sleep can facilitate learning.

## Conclusions

In summary, we investigated differences in learning a functional lower extremity motor skill between mildly impaired stroke survivors and neurologically intact individuals. We found that neurologically intact individuals showed greater motor performance with practice as compared to stroke survivors, but stroke survivors showed greater offline learning than neurologically intact individuals. These findings lend important insights into how stroke affects the learning process and may have potential implications for gait rehabilitation after stroke.

## Data Availability

No datasets were generated or analysed during the current study.

## Abbreviations

DOF: Degree of Freedom
D1: Day 1
D2: Day 2
Tr: Training
